# Supplementary material for: FERN – a Java framework for stochastic simulation and evaluation of reaction networks
Source: BMC Bioinformatics. 2008 Aug 29;9:356. doi: 10.1186/1471-2105-9-356 (PMC2553347; doi:10.1186/1471-2105-9-356)
Supplement: Additional file 1 — FERN distribution, Version 1.3. This archive contains the FERN source code and binaries as well as documentation and example models in FernML and SBML. [file 1471-2105-9-356-S1.zip › fern/doc/javadoc/fern/network/sbml/class-use/SBMLPropensityCalculator.html]

Uses of Class fern.network.sbml.SBMLPropensityCalculator


---


|  |  |  |
| --- | --- | --- |
| **Overview** \| **Package** \| **Class** \| **Use** \| **Tree** \| **Deprecated** \| **Index** \| **Help** | |  |
| PREV   NEXT | **FRAMES**    **NO FRAMES**     **All Classes** |  |


---


## **Uses of Class fern.network.sbml.SBMLPropensityCalculator**

| Packages that use SBMLPropensityCalculator | |
| --- | --- |
| **fern.cellDesigner** |  |

Uses of SBMLPropensityCalculator in fern.cellDesigner

| Subclasses of SBMLPropensityCalculator in fern.cellDesigner | |
| --- | --- |
| `class` | `CellDesignerPropensityCalculator`             Propensity calculator which is used for `SBMLNetwork`s. |

---


|  |  |  |
| --- | --- | --- |
| **Overview** \| **Package** \| **Class** \| **Use** \| **Tree** \| **Deprecated** \| **Index** \| **Help** | |  |
| PREV   NEXT | **FRAMES**    **NO FRAMES**     **All Classes** |  |


---
